# EPIC, Scottish Government's Centre of Expertise in Animal Disease Outbreaks: A Model for Provision of Risk-Based Evidence to Policy

**DOI:** 10.3389/fvets.2020.00119

**Published:** 2020-03-05

**Authors:** Lisa A. Boden, Sheila Voas, Dominic Mellor, Harriet Auty

**Affiliations:** ^1^Global Academy of Agriculture and Food Security, The Royal (Dick) School of Veterinary Studies and The Roslin Institute, Midlothian, United Kingdom; ^2^Animal Health and Welfare Division, Scottish Government, Edinburgh, United Kingdom; ^3^School of Veterinary Medicine, College of Medical, Veterinary and Life Sciences, University of Glasgow, Glasgow, United Kingdom; ^4^Epidemiology Research Unit, Scotland’s Rural College (SRUC), Inverness, United Kingdom

**Keywords:** risk-based evidence, animal health, contingency planning, disease outbreaks, risk communication

## Abstract

EPIC, Scottish Government's Centre of Expertise on Animal Disease Outbreaks, offers a successful and innovative model for provision of scientific advice and analysis to policy-makers in Scotland. In this paper, we describe EPIC's remit and operations, and reflect on three case studies which illustrate how the Centre of Expertise Model provides risk-based evidence through rapid access to emergency advice and analyses, estimating disease risks and improving disease detection, assessing different disease control options, and improving future risk resilience. The successes and challenges faced by EPIC and its members offer useful lessons for animal health researchers and authorities, working in contingency planning for animal health security in other countries.

## Background

Global challenges, such as animal disease outbreaks, are complex multi-faceted problems which demand cross-cutting interdisciplinary collaboration to find scientific and technical solutions which also take into consideration the political and societal dimensions of these events. In Scotland, the Government has invested in four Centre of Expertise models of science-policy exchange to support evidence-based decision-making for environmental, plant and animal/public health risks^1^. EPIC, Scottish Government's Centre of Expertise on Animal Disease Outbreaks (www.epicscotland.org), is funded to develop and provide research capacity to assist in the prevention of, preparation for and eradication of important notifiable animal diseases.

EPIC has been funded by the Rural and Environmental Science and Analytical Services (RESAS) of the Scottish Government since 2006 as a collaborative interdisciplinary research consortium between major scientific research institutions that focus on animal health in Scotland, including both universities and other research providers^2^. The consortium aims to foster a culture in which researchers from different disciplinary and professional domains (i.e., veterinary medicine, epidemiology, genetics, physics, mathematics, statistics, social science, and economics) come together to address policy-relevant questions in “peace-time” as well as during animal disease emergencies.

The original rationale for EPIC was based on the need to improve scientific capacity to respond to animal disease risks and threats which have the potential to cause significant socio-economic harm to the UK. The demand for this capacity was writ large after the Foot-and-Mouth Disease outbreak in 2001 (1). The first important test for EPIC occurred not long after, when in 2007 it was requested by Scottish Government to provide evidence to underpin negotiations with local stakeholders and the European Commission to reopen livestock markets after FMD was detected in England (2). The response to this request helped forge EPIC's reputation for delivering robust, timely policy-relevant outputs in anticipation of, and during, disease outbreaks. This, in combination with EPIC's explicit consideration of best-practice at the science-policy interface (3), resulted in UK-wide recognition of EPIC as “a good model of how to secure the best available scientific advice to inform government policy on reducing the impact of animal disease outbreaks (4).”

In this paper, we describe the EPIC remit and, through a series of case studies, illustrate its operational (Case Study 1), tactical (Case Studies 2A and 2B) and strategic work (Case Study 3). We conclude with a discussion about the opportunities and challenges of this exemplar model for the provision of scientific and other interdisciplinary research evidence and expertise for policy.

Case Study 1 **Estimating risks.**

**Contingency planning for FMD disease outbreak response**

To ensure business continuity and avoid economic losses in the event of future outbreaks, and to respond to SG queries about whether countryside closures were proportional to the risk, EPIC veterinary epidemiologists conducted veterinary risk assessments (VRAs) to assess the risks of recreational activities requiring access to the countryside during an outbreak of FMD. VRAs were developed to estimate the risks associated with 12 activities including walking, cycling, canoeing, fishing, horse riding, staging events on agricultural land, stalking deer and shooting birds, which necessitate access to the countryside in an outbreak (5, 6). The VRAs were assessed by SG and the UK National Experts Group on FMD and shared with other risk assessment teams as a model of good practice in outbreak preparedness. It is anticipated that these VRAs would help to avoid costs associated with the collapse of rural economies and tourism observed during the FMD outbreak in UK in 2001, due to “the countryside being closed” for disease control purposes, which reached £3billion (7).

Case Study 2A **Informing risk management.**

**Informing options for FMD control**

In response to the FMD outbreak in 2007, EPIC developed animal movement models to provide Scottish Government with evidence to underpin a decision to reopen livestock markets (2). In subsequent years, EPIC models have been developed to explore cost-benefits of alternative FMD contingency plans specified under EU legislation (Directive 90/423/EEC)^3^, including a reactive vaccination-to-live policy targeting cattle in Scotland. EPIC's analyses highlighted the potential for cost-savings in large (but not small) outbreaks when vaccination is used (8–11). Further, they quantified the negative impact of suboptimal vaccine dose availability and resultant stocking delays on outbreak control costs. EPIC's assessment of the role of livestock haulage vehicles on the spread of diseases has demonstrated the importance of this route of transmission on the spread of FMD and other diseases and highlighted the need to improve cleaning & disinfection protocols in the UK. EPIC scientists estimated that sharing haulage vehicles limited the efficacy of the standstill regulation that was put in place to prevent widespread FMD outbreaks, resulting in a 10-fold increases in the size of the largest outbreaks.

Case Study 2B **Informing risk management.**

**Transmission and tracking of Bovine Viral Diarrhea: The value of endemic disease models to inform exotic disease preparedness**

Although EPIC's principal focus is on preparedness for, and response to, exotic animal disease outbreaks, endemic disease models for livestock can also offer valuable insights into the interaction between infectious pathogens and various animal species within a local context and enhance EPIC's capability and capacity to respond quickly and effectively in the event of an emergency. The Scottish BVD Eradication Scheme has provided EPIC with a unique opportunity in this regard. EPIC scientists, in collaboration with stakeholders (Biobest laboratories, SAC consulting), sequence BVDV isolates obtained from animal samples to inform the latter stages of the Scottish eradication campaign. Over two thousand samples have been archived and genotyped, providing a reference database for Scotland. Preliminary phylodynamic analysis demonstrates multiple BVDV strain movements between Scotland and the rest of GB. Synthesis of this work with EPIC's experience and familiarity with working with data rich mechanisms of disease spread, such as animal movements, together with insights into farmer and stakeholder experiences (12) provides an important resource that will improve our understanding of BVDV transmission and should inform the final stages of Scotland's BVDV eradication policy (13). EPIC scientists' experience with the methodologies for integrating phylodynamics with other data types for endemic disease provide important resilience in responding to exotic disease outbreaks where similar approaches are valuable.

Case Study 3 **Improving future risk resilience.**

**Anticipating the future of veterinary surveillance in Scotland**

EPIC has a strategic research portfolio which includes participatory foresighting activities (such as scenario planning) led by multidisciplinary, multi-partner teams of researchers, decision-makers and practitioners from different disciplinary domains. Scenario planning is a formal approach used by the private and public sectors and academics (in the social science disciplines in particular) to structure thinking around long-term planning in response to uncertainty. In 2017, the outputs of scenario planning work coincided with a disruptive political shock: the decision of UK to leave the European Union, known as Brexit (14). The scenarios generated from the workshop were co-produced with stakeholders and later shared with the British Veterinary Association Surveillance Working Group; key findings were presented to Scotland's Strategic Management Board for Veterinary Surveillance, the Scottish Futures Group and the British-Irish Parliamentary Assembly Committee inquiry into the implications of Brexit for the agri-food sector. Importantly, the scenarios offer an opportunity for a positive and strategic feedback loop within EPIC to “future-proof” its programme of research and tailor it to anticipate and adapt to future possibilities and uncertainties.

## The Epic Model for Providing Risk-Based Evidence for Policy

EPIC comprises 40 or so scientists who work as part of the EPIC team either full time, or part-time alongside other research and other commitments. The multi-disciplinary expertise of EPIC's members means that it has the capacity for delivering interdisciplinary research to policy-makers to address questions which range from the very applied (e.g., operational or tactical decisions regarding disease control) to the very strategic (e.g., foresighting activities, research and development of innovative methodologies). EPIC has a programme of research agreed with government, to be conducted when no disease outbreak is occurring. This work programme is proposed at the start of each 5-years funding cycle, but is reprioritized as necessary to respond to requests from Scottish Government for specific analyses. In the event of an outbreak, as many EPIC scientists as are required convert to work which informs the outbreak response. The ability to provide a rapid response to emergency outbreak events is facilitated by trusted partnerships between consortium members and Scottish Government veterinarians, scientists and policy officials, and has been underpinned by sustained funding over multiple policy-cycles. The latter has been essential to build meaningful, long-lasting relationships with policy-makers. EPIC's activities at the science-policy interface have been strengthened by integration of EPIC scientists at Government-facilitated stakeholder group meetings to foster knowledge exchange with industry leads and the public. Explicit engagement between EPIC scientists, the Animal and Plant Health Agency (APHA) and Department for the Environment, Farming and Rural Affairs (Defra) also occurs at a UK level to ensure that EPIC's work adds value, avoids unnecessary duplication and is complementary to UK priorities. The relationships between GB and Scottish disease control structures are outlined in the Scottish Government Exotic Disease Contingency Framework Plan [(15), p. 30].

## Research Priorities

EPIC's research priorities align to four strategic foci which are important to Scottish Government and Defra:

1. Risk communication: Providing rapid access to emergency advice and analyses in the event of disease outbreaks, and knowledge exchange.
2. Estimating risks: Characterizing disease risks and improving disease detection.
3. Informing risk management: Assessing different disease control options.
4. Improving future risk resilience: Developing advice on challenges and opportunities presented by local and global societal, technical, economic, environmental, and political developments.

### Risk Communication: Rapid Access to Emergency Advice and Analyses

Academic researchers can struggle to engage with policy through a lack of understanding of how policy-making works, or a lack of ability to communicate science in the most effective way for policy-makers (16). Similarly, policy-makers may not access relevant evidence for policy (or request such evidence to be provided) because they do not know the appropriate academics to approach or the correct questions to ask. Furthermore, there can be a disconnect between the temporal scales of traditional academic research which often looks to the future, and policy need which is often “here and now.” EPIC has addressed this potential dissonance through its investment in experienced knowledge brokers who are embedded in both academia and the SG Animal Health and Welfare Division (AHWD) policy environment to ensure rapid and effective communication across the science-policy interface within and outwith disease emergencies (3). EPIC members work alongside policy-makers in AHWD offices to facilitate effective science-policy translation and knowledge exchange both in “peace-time” and in disease emergencies. These knowledge-brokering roles have been an effective means of communication and co-construction of policy-relevant scientific endeavors. The emphasis placed on understanding animal health policy and governance as a means to improving delivery of relevant scientific evidence, has enabled mutual understanding and trust to grow between the scientific and policy “poles” of EPIC's business. In doing so, there is greater appreciation, on both sides, of the other's pressures, abilities and needs, and the properties of what will be useful outputs. Investment in this science-policy interface has resulted in an agile research model, which enables researchers to navigate successfully between operational and tactical policy-responsive work and longer term strategic and other “blue-sky” research. The former forms the basis of advice to policy-makers whilst the latter is essential to sustain the experience-base, quality and credibility of the science available to inform policy.

### Estimating Risks: Characterizing Disease Risks and Improving Disease Detection

EPIC has dedicated resources to improving preparedness and outbreak response for exotic diseases such as Foot-and-Mouth disease, Highly Pathogenic Avian Influenza, and African Swine Fever via epidemiology, economics and risk assessment (Case Study 1) and is one of few non-government members with representation on the UK's National Emergency Epidemiology Group (formed during exotic disease outbreaks to provide epidemiological input on the determinants and distribution of disease to inform decisions on disease control) and the “5 Nations Veterinary Risk Assessment (VRA) forum” which includes leaders from all relevant agencies and governments from England, the other UK devolved administrations and Republic of Ireland.

EPIC members work with animal and human health surveillance data providers in Scotland to add value to existing data collection systems, develop methodologies to analyse and integrate surveillance datasets and develop risk-based approaches to surveillance to improve disease detection. Horizon scanning tools have been developed in collaboration with Defra to monitor salient disease threats—in particular Bluetongue virus (BTv), African Swine Fever (ASF), and Highly Pathogenic Avian Influenza (HPAI) in order to expedite assessments of risks posed by animal import to other livestock (Bessell et al., under review). This work is notable for its cross-sectoral involvement and multi-disciplinary approach which is important for identifying surveillance gaps and future emerging threats, whether in the form of specific pathogens, or vulnerabilities such as industry practices.

### Informing Risk Management: Assessing Different Disease Control Options

EPIC uses epidemiological modeling and economic approaches to assess disease control options, which are ground-truthed through interactions with livestock industry experts (Case Study 2A). Endemic disease models also offer instructive exemplars of how to improve exotic animal disease preparedness (Case Study 2B). EPIC's modeling expertise and experience is therefore always current and routinely adapted to policy- and industry-specific queries which means that there is readiness to respond to new and emergent threats such as Schmallenberg Virus (17) and ASF (18), #muckfreetruck campaign^4^, when/if they occur.

### Improving Future Risk Resilience

Developing advice on challenges and opportunities presented by local and global societal, technical, economic, environmental, and political developments.

EPIC utilizes novel combinatorial approaches, including the application of social science and business management tools such as scenario planning to integrate interdisciplinary expertise and create transdisciplinary solutions [Case Study 3, (14, 19)]. Scenario planning exercises have been held with a wide range of cross-sectoral stakeholders and decision-makers to consider and co-create credible long-term futures in order to enhance opportunities and mitigate challenges to facilitate earlier diagnoses and detection of exotic, endemic, and novel animal and zoonotic diseases in different industry sectors. This participatory method offers an opportunity for inclusive and reflexive approaches which enable up-stream engagement with research beneficiaries. It also enables positive feedback loops within EPIC to “future-proof” risk assessment tools and encourage improved risk communication.

## Challenges and Successes for Epic

**Figure 1:**
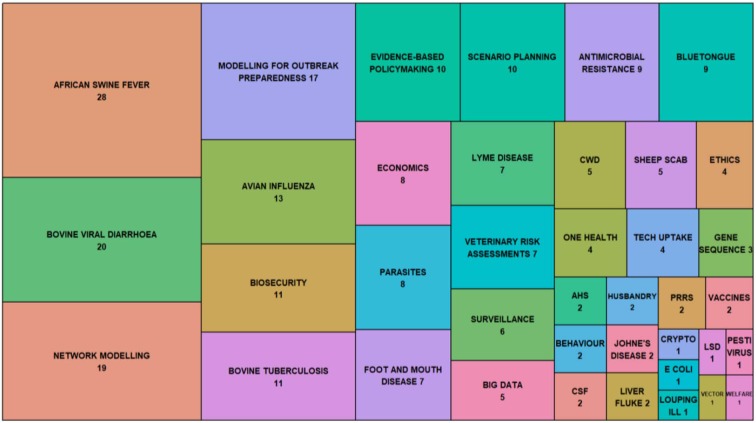
Treemap of a sample of EPIC's knowledge exchange outputs between March 2016 and 2019 to illustrate the range of EPIC work. In total, EPIC researchers recorded 486 KE-related outputs during this time period. In this graph, we present a subset of those that referenced a specific policy focus, animal disease topic or methodology (*n* = 253). These outputs included peer-reviewed publications, policy and research briefs, stakeholder meetings, technical reports for government, conference presentations, and posters. Due to space constraints, some words and terms in the figure have been abbreviated: AHS, African Horse Sickness; Crypto, Cryptosporidiosis; CWD, Chronic Wasting Disease; *E. coli, Escherichia coli*; LSD, Lumpy Skin Disease; PRRS, Porcine Reproductive and Respiratory Syndrome; Tech uptake, Technology uptake; Vector, Vector-borne Disease; Welfare, Animal Welfare.

EPIC illustrates a model of research provision for policy-making that utilizes academic partners, working closely with Government policy-makers, to contribute to evidence-based decision-making. EPIC's continued funding over more than 10 years has provided the opportunity to develop and refine EPIC's remit and operations. As a result, EPIC researchers have been able to deliver tangible policy-relevant outcomes (e.g., Case Studies 1, 2, 3) via a broad range of outputs (Figure 1). EPIC's successes have come from building long-term sustained relationships with policy-makers that allow for meaningful and genuine engagement. The specific impacts of this type of approach are hard to quantify as they go beyond traditional academic metrics (of peer-reviewed publications and patents) and include broader conceptual changes about how the scientists and policy-makers interact, moving further toward a co-production approach (20), illustrated by the case studies presented.

One of the challenges of this model is that academics value quality scientific publications, which take time to develop, whilst policy-makers need timely, trusted information to inform policy decisions. Responding to requests from policy-makers helps academics to produce more impactful science, but does not always lead to the scientific publications that they and/or their employers, desire. Focusing on policy-oriented research can therefore be a barrier to career progression within academic organizations. The increased emphasis on “research impact” that has emerged over the last few years (21) is helpful in promoting the value of academics engaging in policy-oriented work, although it potentially rewards a rather simplistic view that research leads directly to policy, rather than a more nuanced co-production approach (20). A real benefit of initiatives such as EPIC is in building up a cohort of personnel with the technical capacity to provide analyses in outbreak situations, but careful consideration is needed to ensure the structures do not inadvertently inhibit personal career development.

## Conclusions

Risks to animal health and welfare have changed rapidly, and will continue to evolve and become increasingly complex in future. The increasing liberalization of trade combined with a changing climate has resulted in an increase in the velocity and volume of people, animals and animal products moving around the globe (22). This, in combination with the translocation of non-native disease hosts and vectors as a result of climate change and urbanization, creates the potential for new and (re)emerging transboundary disease outbreaks of significant socio-economic importance (22). These risks are illustrated by the current threat of ASF. This lethal pig disease has already taken hold in Europe and Asia, and is having far-reaching effects in global health and food security, that are likely to be felt long after the initial outbreak has subsided.

The current global ASF outbreak illustrates the importance of coordinated interdisciplinary efforts which consider systems-approaches to animal disease preparedness. EPIC's current governance and organizational structure offers a framework for the effective deployment of interdisciplinary capabilities in the natural sciences, social sciences, economics and the humanities, in an enduring and resilient way to support a coordinated vision for animal health policy through appropriate risk prioritization, analysis and communication. In the UK, and in particular in Scotland, this approach has helped to reduce research wastage through avoidance of duplication of efforts, build research skills and capacity, and generate targeted evidence to improve cost-effective interventions ensuring the long-term resilience of animal health policy and food security.

## Data Availability Statement

Datasets are in a publicly accessible repository. The datasets generated for this study can be found at https://figshare.com/articles/Treemap_data/11918967.

## Author Contributions

LB was responsible for the conception of the paper. LB and HA wrote sections of the manuscript. All authors contributed to manuscript revision, are accountable for the accuracy and integrity of this work and have approved the final manuscript for publication.

### Conflict of Interest

The authors declare that the research was conducted in the absence of any commercial or financial relationships that could be construed as a potential conflict of interest.

## Footnotes

^1^EPIC, Scottish Government's Centre of Expertise on Animal Disease Outbreaks; ClimateXChange, Scotland's Centre of Expertise on Climate Change; Crew, Scotland's Centre of Expertise for Waters; Centre of Expertise on Plant Health.

^2^The University of Edinburgh [The Global Academy of Agriculture and Food Security, Royal (Dick) School of Veterinary Studies and The Roslin Institute], The University of Glasgow, Scotland's Rural College, Moredun Research Institute, The James Hutton Institute and Biomathematics and Statistics Scotland.

^3^Council Directive 90/423/EEC of 26 June 1990 amending Directive 85/511/EEC introducing Community measures for the control of foot-and-mouth disease.

^4^#MuckFreeTruck. Available at: http://www.npa-uk.org.uk/_MuckFreeTruck_campaign_launched_to_encourage_better_lorry_washing.html.
